# Observation of the Zero Doppler Effect

**DOI:** 10.1038/srep23973

**Published:** 2016-04-05

**Authors:** Jia Ran, Yewen Zhang, Xiaodong Chen, Kai Fang, Junfei Zhao, Hong Chen

**Affiliations:** 1 Tongji University, Shanghai, 200092, China; 2 University of Electronic Science and Technology of China, Chengdu, 610054, China; 3 Queen Mary University of London, E1 4NS, UK

## Abstract

The normal Doppler effect has well-established applications in many areas of science and technology. Recently, a few experimental demonstrations of the inverse Doppler effect have begun to appear in negative-index metamaterials. Here we report an experimental observation of the zero Doppler effect, that is, no frequency shift irrespective of the relative motion between the wave signal source and the detector in a zero-index metamaterial. This unique phenomenon, accompanied by the normal and inverse Doppler effects, is generated by reflecting a wave from a moving discontinuity in a composite right/left-handed transmission line loaded with varactors when operating in the near zero-index passband, or the right/left-handed passband. This work has revealed a complete picture of the Doppler effect in metamaterials and may lead to potential applications in electromagnetic wave related metrology.

The Doppler effect is a common phenomenon that the relative motion between a source and the detector causes a frequency shift in the received wave signal. In our daily life experience, i.e. in the normal dispersion media with both positive permittivity *ε* and permeability *μ*, an approaching source increases the received signal frequency, whereas a receding source decreases it.

Recently, the inverse Doppler effect, that the frequency decreases when the source is approaching the detector whereas increases when it is leaving the detector^1^, was experimentally demonstrated by Seddon and Bearpark^2^ and others^3^,^4^,^5^,^6^,^7^,^8^,^9^ in the so-called left-handed (LH) metamaterials^10^,^11^,^12^,^13^,^14^,^15^,^16^,^17^. The mechanism for this abnormal phenomenon is that the group velocity and phase velocity are anti-parallel in such left-handed materials, deriving from that the permittivity *ε* and permeability *μ* are both negative (double negative materials, DNG). The rotational Doppler effect in left-handed metamaterials is also discussed in ref. 18.

A natural question is whether we can find a medium in which the zero Doppler effect, that is, no frequency shift irrespective of the relative motion between the source and the detector, can be observed experimentally. This question has motivated us to study the Doppler effect in a metamaterial with both permittivity and permeability near zero, referred as (near) zero-index metamaterial (ZIM)^19^,^20^,^21^,^22^,^23^,^24^. It is demonstrated that the wave phase velocity is infinite while there is still electromagnetic (EM) wave transfer inside the zero-index metamaterial^25^,^26^, as shown in Supplementary Movie 1.

We have realized the zero-index metamaterial on a tunable composite right/left-handed transmission line (CRLH TL)^26^,^27^,^28^,^29^ loaded with varactors^3^,^30^. The constructed CRLH TL exhibits in turn a left-handed passband, a stopband and a right-handed (RH) passband over the operating frequency band^27^,^28^. By choosing the proper component parameters on the CRLH TL (see details in Methods), the stopband can be narrowed down to vanish at a frequency at which both permeability and permittivity are near zero, referred as a balanced CRLH TL^17^. Hence, the (near) zero-index metamaterial is obtained at this frequency.

In order to conduct the Doppler effect experiment, we need to apply different bias voltages on the varactor loaded on the CRLH TL to switch the transmission characteristics of the CRLH transmission units between the (near) zero-index passband and a stopband by a digital circuit referred as the reflective interface controller^3^. In our Doppler effect experiments, instead of moving the source or the detector, we have created a moving interface to reflect the incoming RF signal. The reflective interface is formed by setting the left section of CLRH TL units in passband and the right section of units in band gap (stopband). When switching the transmission characteristic of the unit next to the reflective interface from band gap to passband, the reflective interface can be moved one unit rightward. Switching the transmission characteristics of the units next to the reflective interface in succession, a moving reflective interface can be achieved on the transmission line. The reflective interface could be considered moving successively when the wavelength is considerably larger than the length of the transmission unit. Depending on the time interval of the alternation of adjacent units’ bias voltages, the moving velocity of the reflective interface *v*_*s*_ can be varied through the reflective interface controller. The RF signal is injected through a circulator into this CRLH transmission line when the passband is set at zero-index point as shown in Fig. 1.

## Results

### Characteristics of the tunable composite right-left handed transmission line

The unit of the composite right-left handed transmission line is composed of microstrip line loaded with two 19 pF capacitors in series and one varactor NXP BB131 in parallel, as shown in Fig. 2a. Once the bias voltages changes, the capacitances of the varactor would vary - leading to the variation of dispersion curve. The theoretical dispersion curve of the transmission line can be computed by using ABCD matrixes (See details in Supplementary Text). The relative permittivity and permeability are:


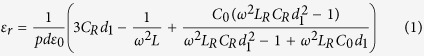



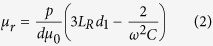


where *p*, the structure constant, is 4.05 in our case. *ε*_0_, *μ*_0_ are permittivity and permeability in vacuum separately. *C*_*R*_, *L*_*R*_ are the distributed capacitance and inductance of the transmission line, *C*, *C*_0_ are the capacitance of the loaded series capacitor and the varactor separately. *d*, *d*_1_ are the lengths of unit and the microstrip line shown in Fig. 2a, see details in Methods.

The capacitance of the varactor *C*_0_, will vary with the bias voltage. Therefore, by choosing a proper series capacitor *C* and bias voltage, one can make both permittivity and permeability near zero, leading to a zero-index transmission line and zero wave vector. Meanwhile, the negative and positive permittivity and permeability can also be obtained by choosing different operating frequencies.

Figure 2b is the comparison between theoretical and experimental dispersion curves of the balanced transmission line. The inset is the zoom-in of the linear dispersion at zero-index frequency which is emphasized in a green ellipse. The experimental zero-index frequency is achieved at *f*_*Z*_ = 852 MH_Z_, slightly lower than the theoretical one *f*_*Z′*_ = 879 MH_Z_. The phase velocity is shown in Fig. 2c, which turns to be very large at the zero-index frequency. The propagation of wave at *f*_*Z*_ inside the transmission line is shown in Supplementary Movie 1, in which one can see there is no phase variation inside the CRLH TL, while the energy keeps flowing through it.

Figure 2d illustrates the experimental transmission characteristics (in terms of |*S*_21_|) of the CRLH transmission line under two bias voltages of 16 V (red solid line) and 0 V (blue dotted line). We can see that the transmission sustains across a wide frequency band, covering left-handed region, zero-index frequency and right-handed region when the transmission line is balanced under 16 V bias voltage (see Supplementary Fig. 2b). There is around 10 dB loss, which is mainly due to the reflection at the input port (see Supplementary Fig. 3b). When the bias voltages are turned off to 0 V, the capacitance of the varactors increases and the original passband becomes a band gap (stopband). Therefore, a reflective interface can be formed between units set in passbands (16 V) and band gap (0 V).

### Theoretical Doppler frequency shifts

As a unique character of zero-index metamaterial, electromagnetic wave inside such ZIM has an infinite long wavelength, exhibiting a constant spatial distribution that varies temporally while propagating through the ZIM (see details in Supplementary Text and Movie 1)^25^, resulting in that the detector can not “sense” any compression or stretch of the wave front. As a result, the zero Doppler effect can be observed under this condition.

The Doppler effect can be governed by the following equation between the frequencies of the incident wave *f*_*i*_ and reflected wave *f*_*r*_^2^:


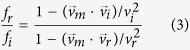


where 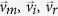 are the velocities of the moving reflective interface, incident wave and reflected wave separately. The velocities are positive when moving away from the source. In our work, the phase velocities of the incident and reflected wave are near equal (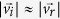) in the range of Doppler shifts (see more in the Supplementary Text).

At the zero-index frequency *f*_*z*_, the refractive index and propagation constant are both close to zero. Hence the phase velocity at *f*_*z*_ is infinite according to its definition (
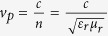
, where *c* is the wave speed in free space), shown in Fig. 2c. Therefore, equation (3) falls to:


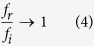


when *f*_*i*_ = *f*_*z*_.

Equation (4) shows that the reflected signal frequency would always keep the same with the incident signal frequency, no matter how fast the reflective interface moves, which is called the zero Doppler effect.

### Measured zero Doppler shifts

In order to demonstrate the zero Doppler effect in experiment, the spectra of the reflected signal with incident frequencies near the zero-index frequency are displayed in Fig. 3. Spectra at left side are under the condition that the reflective interface is approaching the source at four different velocities, while the ones at right side are under the condition that the reflective interface is receding at the same speed as the left ones.

When the incident frequency is set either below the zero-index frequency (*f*_*L*_ = 690 MH_Z_) in Fig. 3a or above the zero-index frequency (*f*_*R*_ = 990 MH_Z_) in Fig. 3c, there appear two major peaks on the spectra at each velocity of the reflective interface: the higher peak corresponding to the incident frequency owing to the reflection at the input port, while the lower peak corresponding to the frequency of shifted components, i.e. the (inverse) Doppler shifts. However, when the signal is injected into the CRLT transmission line at the zero-index frequency (*f*_*Z*_ = 852 MH_Z_) in Fig. 3b, there only appear one major peak on the spectra, corresponding to the incident frequency, irrespective of the motion of the reflective interface. As a result, we can conclude that zero Doppler shift was observed at the zero-index frequency.

Figure 4 shows a complete picture of the Doppler effects (normal, inverse and zero shifts) when the EM wave travels in this balanced transmission line, depending on the moving velocity of the reflective interface and frequency. The surface is the theoretical result computed by using Equation (3) while the lines with markers are measured ones (yellow, green are the inverse, normal Doppler shifts separately). Line a and c are the measured inverse and normal Doppler shifts at 570 MHz and 1.07 GHz specifically, whose spectrum are shown in Supplementary Fig. 6. The near zero frequency shifts at zero-index frequency are observed in the resolution of 0.2 MHz, denoted by red dotted line b. We can see that the surface is twisted around the zero-index frequency and the theoretical slope of line b at 852 MHz is near zero, which means that, no matter which direction (*v*_*s*_ > 0 or *v*_*s*_ < 0) and how fast the reflective interface moves, there would be no frequency shift. That is what we called zero Doppler effect.

## Discussion

The zero Doppler effect is observed for the first time in experiment on a zero-index metamaterial based on a tunable composite right/left-handed transmission line loaded with the varactors. As a unique character of the zero-index material, the wave propagates through it without any phase change, leading to neither phase compression, nor phase stretch when there is a relative motion between the signal and the detector. As a result, there would be no Doppler shift no matter how the relative motion is.

The most crucial parts of our experiment are the realization of the zero-index metamaterial and the formation of reflective interface on this CRLH transmission line, which can move at a certain velocity controlled by an electrical circuit, leading to a relative motion between the source and the detector. The normal Doppler effect or the inverse Doppler effect can also be realized on the CRLH transmission line when the incident frequency falls into the right-handed passband or the left-handed passband respectively. The maximal frequency shift is mainly limited by the performances of the components on the digital switching circuit, which lead to the finite rising and falling edges (~10 ns for each) of the pulse signal. As a result, the internal of the bias voltages’ rising or falling edges would be no shorter than 20 ns. Utilizing high performance components can therefore increase the maximal Doppler shift and the resolution. It is worth mentioning that zero Doppler effect observed in the zero-index metamaterials is different from the concept of ‘zero Doppler effect’ in radar technology, which stands for a technique to compensate the normal Doppler frequency shift. The wave in the near zero-index medium has been shown no frequency shifts irrespective of the relative motion between the source and the detector. It is expected that our work may attract more research interest in exploiting zero-index metamaterials and lead to potential applications in EM wave related metrology.

## Methods

### Fabrication of the 1D tunable composite right-left handed transmission line (CRLH TL)

The tunable composite right/left-handed transmission line (CRLH TL) is fabricated on the FR4 dielectric slab with permittivity *ε*_r_ = 4.75, thickness *h* = 1.6 *mm*. The microstrip lines are made of copper with width *w* = 3 *mm*, lengths *d*_1_ = 3.6 *mm*, *d*_2_ = 1.8 *mm*. The unit of the transmission line is loaded with two capacitors *C* = 19 pF in series, one inductance *L* = 10 nH and a varactor BB131 in parallel. The distributed capacitance and inductance are *C*_*R*_ = 1.2789 × 10^−10^ F and *L*_*R*_ = 3.1978 × 10^−7^ H. The varactor is connected to the ground via hole. Total length of the unit is *d* = 12.37 mm, shown in Supplementary Fig. 1a.

### Setting up of the CRLH TL, inverse and normal Doppler shift measurements

**Figure 1:**
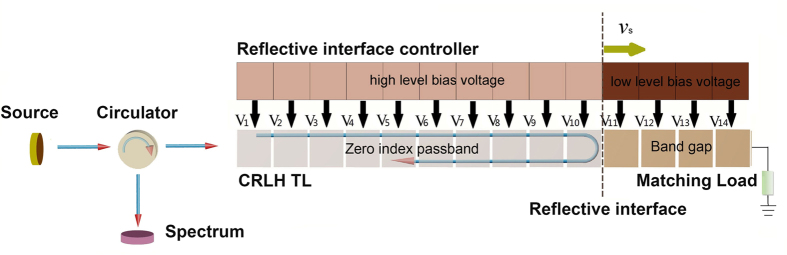
Schematic of the set-up to realize the zero, inverse and normal Doppler Effect. The whole experimental set-up. V_*i*_(*i* = 1, 2…14) are the bias voltages provided to the CRLH TL units. The TL units in gray are set in zero-index passband (can also be LH, RH at different frequencies) while units in dark brown are set in band gap. *v*_s_ is the moving velocity of the reflective interface.

**Figure 2:**
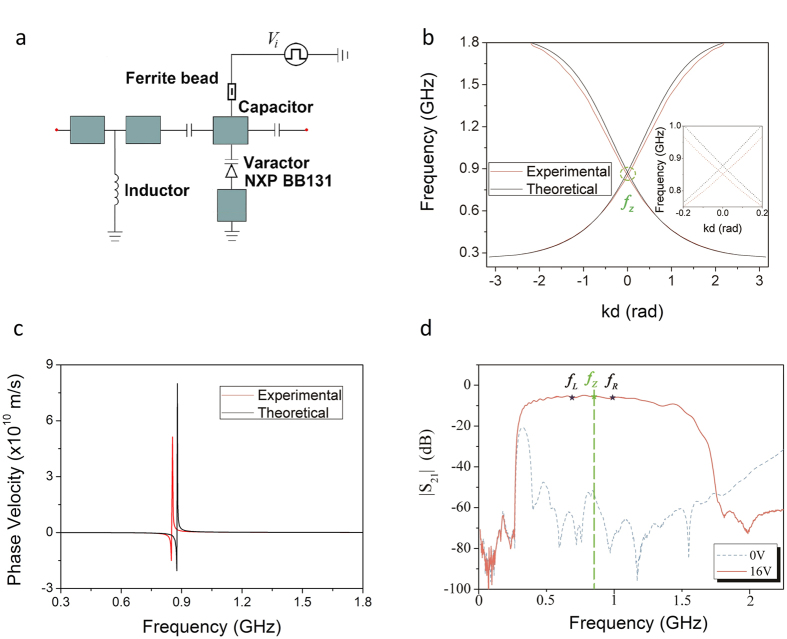
Transmission characteristics of the transmission under different bias voltages. (**a**) Schematic of the CRLH TL unit. See more details in Methods and Supplementary Text. (**b**) The theoretical dispersion curves derived by ABCD matrix method and experimental dispersion curves of the balanced CRLH TL, the inset is the zoom-in of curves inside the green ellipse. *f*_*Z*_ is the experimental (near) zero-index frequency 852 MHz. (**c**) The theoretical and experimental phase velocities obtained from Fig. 2b. (**d**) |S_21_| of the CRLH TL under 16 V (red solid lines) and under 0 V (blue dotted line). *f*_*L*_, *f*_*R*_ are frequencies 690 MHz and 990 MHz specifically. The former is frequency set in left-handed passband while the latter is set in right-handed passband. Green dotted line indicates the zero-index frequency.

**Figure 3:**
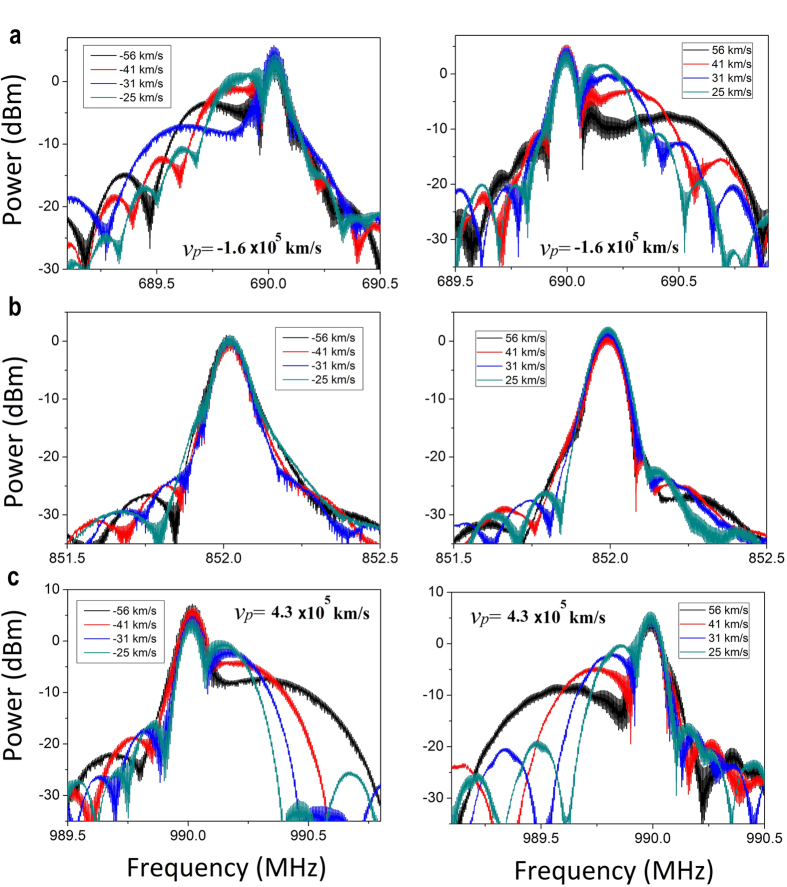
Spectra of the experimental inverse, zero and normal Doppler effects. (**a**–**c**) Spectra of the reflected wave at three frequencies: (**a**) *f*_*L*_ = 690 MH_Z_, (**b**) *f*_Z_ = 852 MH_Z_, and (**c**) *f*_*R*_ = 990 MH_Z_ with the same speed of the reflective interface. The spectra at left side are under the condition that the interface is approaching the source while the spectra at right side are under the condition that the interface is moving away from the source.

**Figure 4:**
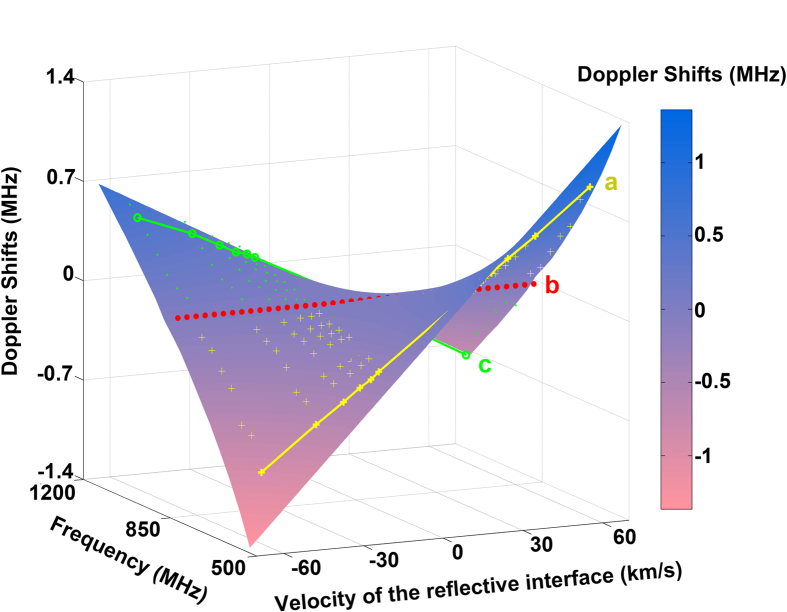
Comparison between the theoretical and experimental Doppler shifts. The surface is the theoretical shifts based on Equation (3) while the markers are experimental shifts, with yellow cross-shaping markers as inverse Doppler shifts and green dots as normal Doppler shifts. Lines a, c are experimental shifts picked to underline the inverse and normal Doppler shifts separately. Line b is the measured zero Doppler shifts in the resolution of 0.2 MHz.

The Agilent network analyzer N5222A is used to test the transmission characteristic of the composite right-left handed transmission line. All the units of the CRLH TL are supplied the same bias voltage in this test. The set-up for observation of inverse Doppler shifts, shown in Fig. 1, is composed by the Agilent vector signal generator E8267D, a circulator with isolation 20 dB, the Agilent signal analyzer N9020A, the 1D tunable CRLH TL, a 50 Ω matching load and the reflective surface controller. The *Gate* function of the spectrum analyzer is used to select certain time domain when the zero/inverse/normal Doppler shift is happening by inputting the voltage signal of the first unit of the reflective surface controller to the analyzer as the trigger signal and setting the duration of the inverse Doppler shift as the length of the *Gate* function.

## Additional Information

**How to cite this article**: Ran, J. *et al.* Observation of the Zero Doppler Effect. *Sci. Rep.* **6**, 23973; doi: 10.1038/srep23973 (2016).

## Supplementary Material

Supplementary Information

Supplementary Movie 1
