# Supplementary material for: Observation of the Zero Doppler Effect
Source: Sci Rep. 2016 Apr 5;6:23973. doi: 10.1038/srep23973 (PMC4820719; doi:10.1038/srep23973)
Supplement: Supplementary Information [file srep23973-s1.pdf]

Supplementary Materials for

Observation of the Zero Doppler Effect

Jia Ran, Yewen Zhang*, Xiaodong Chen*, Kai Fang, Junfei Zhao, Hong Chen

*Corresponding author. Email: yewen.zhang@tongji.edu.cn (Y. Z.);
Xiaodong.chen@qmul.ac.uk (X. C.)

This PDF file includes:

Supplementary Note

Supplementary Figures 1-6

Supplementary Movie 1 caption

Other Supplementary Material for this manuscript includes the following:

Supplementary Movie 1

Supplementary Note

1. Transmission characteristics of the CRLH TL

The theoretical dispersion line of the transmission line can be computed by using ABCD matrixes. The CRLH TL unit is a cascade network with 8 elements:

1). The transmission matrix of the microstrip line section in dark cyan in Supplementary Fig.1 is:

$$M_1 = \begin{bmatrix} \cos\theta_1 & jZ_0 \sin\theta_1 \\ \dfrac{j\sin\theta_1}{Z_0} & \cos\theta_1 \end{bmatrix}$$

where $\theta_i = \beta d_i, i = 1, 2$, and $\beta = 2\pi f \sqrt{L_R C_R}$, f is the frequency of the incident wave. Z_0 is the characteristic impedance of the transmission line.

2). The transmission matrix of the inductance L is:

$$M_2 = \begin{bmatrix} 1 & 0 \\ \dfrac{1}{j\omega L} & 1 \end{bmatrix}$$

where ω is the circular frequency.

3). The transmission matrix of the capacitor is:

$$M_3 = \begin{bmatrix} 1 & \dfrac{1}{j\omega C} \\ 1 & 1 \end{bmatrix}$$

4). The transmission matrix of the microstrip line section in yellow is:

$$M_4 = \begin{bmatrix} \cos\theta_2 & jZ_0 \sin\theta_2 \\ \dfrac{j\sin\theta_2}{Z_0} & \cos\theta_2 \end{bmatrix}$$

5). The 5th element is composed of the varactor and TL in light green. Their transmission matrices in series are $MVar, MTL$ separately:

$$MVar = \begin{bmatrix} 1 & \dfrac{1}{j\omega C_V} \\ 1 & 1 \end{bmatrix}, MTL = \begin{bmatrix} \cos\theta_2 & jZ_0 \sin\theta_2 \\ \dfrac{j\sin\theta_2}{Z_0} & \cos\theta_2 \end{bmatrix}$$

where C_V is the capacitance of the varactor. It is 1.6 pF when supplied the bias voltage 16 V and 18.7 pF with 0 V at 852 MHz.

The transmission matrix of this cascade in series is

$$P = MVar \cdot MTL \tag{1}$$

Here comes the transmission matrix of the 5th element:

$$M_5 = \begin{bmatrix} 1 & 0 \\ \dfrac{P(2,2)}{P(1,2)} & 1 \end{bmatrix}$$

where $P(i, j)$ is the element in the row *i* and column *j* of P.

Since these 8 sections are cascade connected, we can obtain the final transmission matrix of this unit:

$$M = M_1 \cdot M_2 \cdot M_1 \cdot M_3 \cdot M_4 \cdot M_5 \cdot M_4 \cdot M_3 \tag{2}$$

The dispersion relation of the CRLH TL is:

$$\cos(kd) = \frac{M(1,1) + M(2,2)}{2} \tag{3}$$

where k is the propagation constant of the CRLH TL. $M(i, j)$ is the element in *i*th row and *j*th column of M.

The theoretical dispersions of the CRLH TL loaded with different capacitors when supplied a bias voltage of 16 V are calculated by equation (3), shown in Supplementary Fig.2a. With the increase of the capacitance, one of the cutoff frequencies decreases and the CRLH TL is balanced when loaded with 15 pF capacitor. The corresponding experimental $|S_{21}|$ of the CRLH TL is shown in Supplementary Fig.2b.The green dotted line indicates the (near) zero frequency f_z=852 MHz.

Supplementary Fig.3 shows that reflection coefficient of the stopband is approximately 10 dB higher than the passband's, resulting the more reflection on the reflective interface than on the input of the transmission line. There are also much loss along the transmission line due to the shortcut to the ground.

2. Phase velocity calculation

We can obtain the algebraic solution of the phase velocity and plot it in Supplementary Fig.4. It illustrates that the dispersion of the CRLH TL is linear in the measured Doppler shift rang ($\pm$1.4 MHz) and the velocity varies slightly in this range that the variation of the phase velocities is less than 0.02% of the incident frequency's. Hence we have approximately $|\vec{v_i}| \approx |\vec{v_r}|$, which simplifies the Doppler equation to :

$$\frac{f_r}{f_i} = \frac{v_i^2 - v_m v_i}{v_i^2 + v_m v_i} \tag{4}$$

where $|v_i| = \frac{\omega}{k}$ with its sign depends on whether it is right-handed passband ($v_i > 0$) or left-handed passband ($v_i < 0$).

3. Sets of the reflective interface controller and incident port

With the balanced transmission line, the inverse, zero and normal Doppler effect can be realized at certain frequencies only if we could build a moving reflective interface inside the transmission line. Such reflective interface can be obtained by changing the capacitance of varactors in parallel on the transmission line. Applying different bias voltages for the units of the transmission line, a reflective interface can be formed at the interface between the passband (16 V) and the band gap (0 V). Because the reflective interface controller can only move the rising (falling) edge of the output voltages rightwards, a series of experimental sets should be introduced in order to realize both receding and approaching reflective interfaces. By choosing proper incident direction of wave and turning edge of bias voltages (either rising or falling), the reflective interface can be obtained both approaching and moving away from the source. By choosing the falling (rising) edge as the reflective interface and the wave is incident from the right (left) port, a reflective interface approaching (moving away from) the source is formed, shown in Supplementary Fig.5a (b).

Supplementary Figures

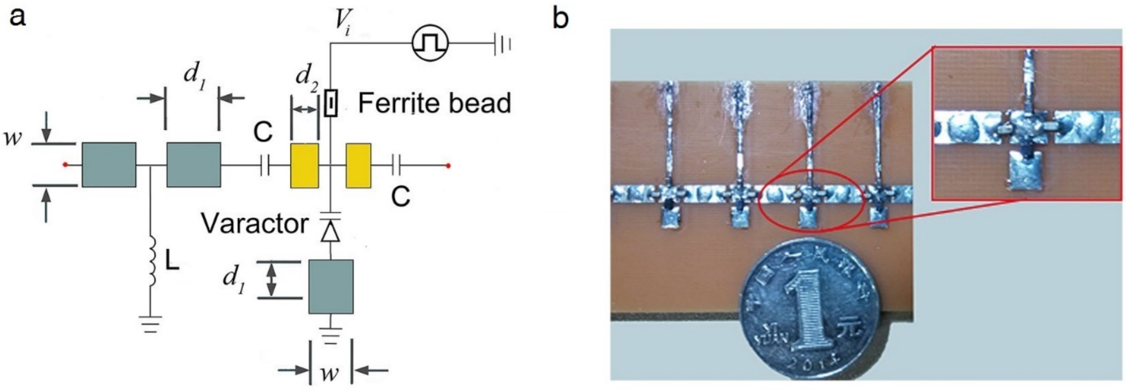

Supplementary Figure 1 | Structure of the unit of the CRLH TL. (a) Schematic of the unit. (b) Photo of a part of the CRLH TL. The inset is the zoom-in of one unit.

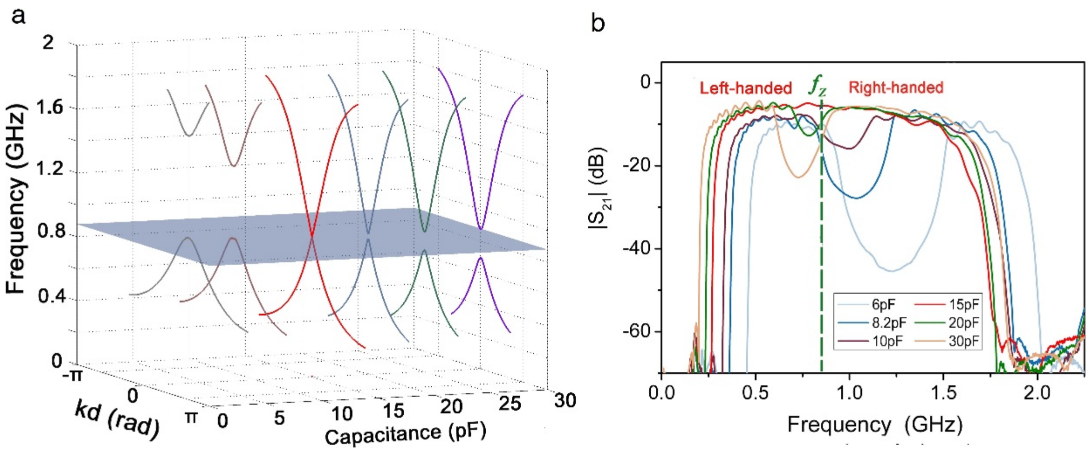

Supplementary Figure 2 | The transmission characteristics of the CRLH TL loaded with different capacitors and supplied with 16 V bias voltage. (a) Theoretical dispersion lines of the CRLH TL. The gray plane underlines frequency 879 MHz which is the theoretical (near) zero frequency. (b) The experimental $|S_{21}|$ of the CRLH TL. The green dotted line indicates the (near) zero index frequency.

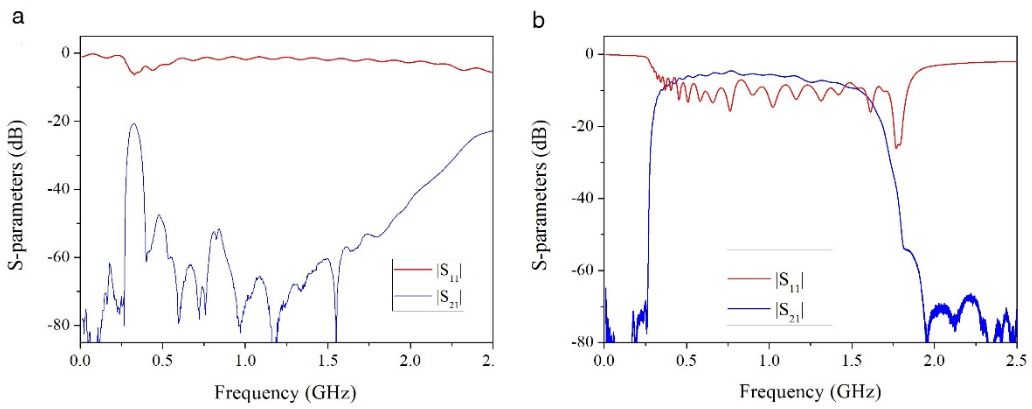

Supplementary Figure 3 | The measured S-parameters of the CRLH TL supplied with 0 V (a) and 16 V (b) bias voltage.

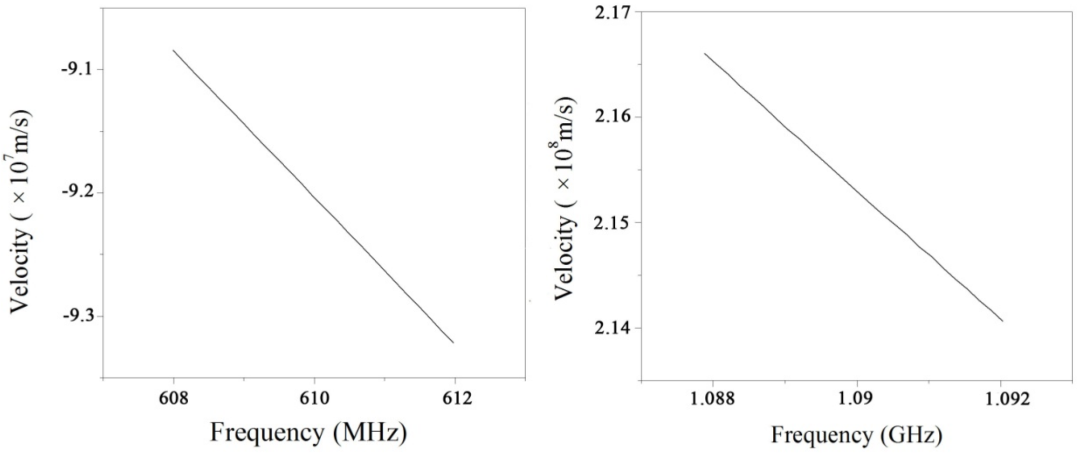

Supplementary Figure 4 | The phase velocities of frequencies in the (abnormal) Doppler shift range with incident frequency 610 MHz (left) and 1.09 GHz (right).

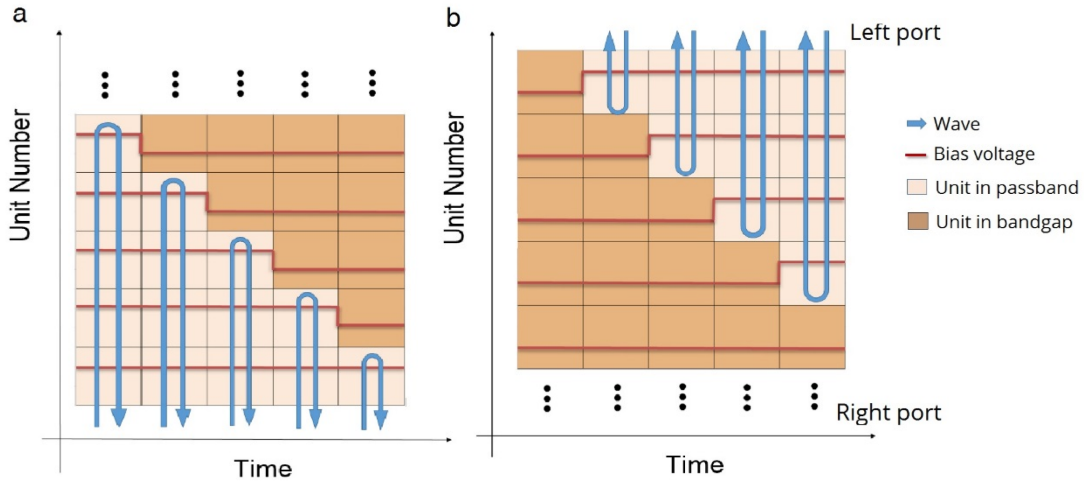

Supplementary Figure 5 | Experimental sets to realize approaching (a) and receding (b) reflective interface.

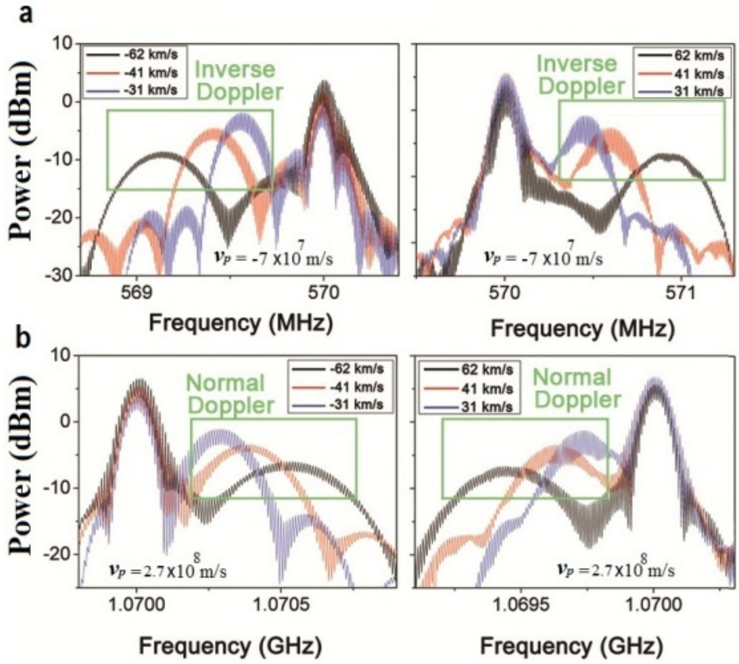

Supplementary Figure 6 | Spectrum of the inverse and normal Doppler shifts at 570 MHz (**a**) and 1.07 GHz (**b**) separately. The phase velocity of the two frequencies are -7×10^{7} m/s and 2.7×10^{8} m/s .

Supplementary Movie 1 Caption

Supplementary Movie 1: Movie of wave distribution inside the (near) zero-index metamaterial. The Microwave & RF Components of CST (Computer Simulation Technology) Studio Suit is used to simulate the wave behavior along the balanced composite right/left-handed transmission line at the simulated zero-index frequency 820 MHz, which is slightly lower than the experimental one 852 MHz due to the dispersions of elements. Movie S1 is the capture of the normal electrical field at 820 MHz, from which we can see that there is no amplitudes' variation along the ZIM section while the wave does propagate through the ZIM.
